# Disparities in Timeliness of Cancer Diagnosis Across a Multi-Site Academic Health System

**DOI:** 10.21203/rs.3.rs-8767825/v1

**Published:** 2026-04-23

**Authors:** Vivian Hoang Tran, Suraj Manohar Rajan, Carol Y. Ochoa Dominguez, Joshua Demb, Humberto Parada, Elena Martinez, Corinne McDaniels, Noe Crespo, Melody K. Schiaffino, Winta Mehtsun, James Murphy, Brenton Rose, Matthew P. Banegas

**Affiliations:** University of California, San Diego; University of California, San Diego; University of California, San Diego; University of California, San Diego; University of California, San Diego; University of California, San Diego; University of California, San Diego; University of California, San Diego; University of California, San Diego; University of California, San Diego; University of California, San Diego; University of California, San Diego; University of California, San Diego

## Abstract

**Background:**

Early cancer diagnosis improves survival and quality of life, yet disparities in stage at diagnosis persist. This study evaluates demographic, clinical, insurance, and neighborhood-level socioeconomic factors associated with late-stage cancer diagnosis within an academic health system.

**Methods:**

We conducted a retrospective cohort study of 27,064 adults diagnosed with breast, colorectal, or lung and bronchus cancer between 2015 and 2025 in the University of California Health System. Late-stage disease was defined as AJCC stage III/IV. Multivariable logistic regression examined associations between late-stage diagnosis and patient characteristics, insurance status, comorbidity burden, and neighborhood socioeconomic measures, including the Area Deprivation Index (ADI), Social Vulnerability Index (SVI), and Healthy Places Index (HPI).

**Results:**

17.6% of patients were diagnosed at a late stage. Cancer type was the strongest predictor, with lung (aOR ≈ 13–14) and colorectal cancer (aOR ≈ 8) associated with higher odds of late-stage diagnosis compared with breast cancer. Residence in medium and high ADI tertiles and Medicaid insurance (OR = 1.16; 95% CI: 1.06–1.28) were associated with higher odds of late-stage diagnosis, while Veterans Affairs coverage was associated with lower odds (OR = 0.76; 95% CI: 0.58–1.01). SVI was not associated with stage at diagnosis, whereas higher HPI scores were modestly protective.

**Conclusion:**

Late-stage cancer diagnosis is driven primarily by cancer type and insurance status, with additional contributions from neighborhood disadvantage.

## Introduction

### Context

Early cancer detection is a fundamental public health strategy recognized by the World Health Organization (WHO) for reducing global cancer morbidity and mortality.^1,2^ Early-stage diagnosis is consistently associated with improved survival, reduced treatment intensity, and better quality of life across cancer types.^2,3^ In the United States (US), sustained declines in cancer mortality since the early 1990s have been attributed in part to advances in screening and early detection, with an estimated 3.8 million cancer-related deaths averted during this period.^3^ Despite these gains, a substantial proportion of cancers continue to be diagnosed at advanced stages, underscoring persistent barriers to timely diagnosis and access to care.^3,4^

Stage-specific survival data reveal that early diagnosis is critical for improving patient outcomes.^2^ For localized cancer, when the disease is confined to its primary site, the 5-year relative survival rates are significantly higher compared to distant (metastatic) disease across the majority of cancer types.^5^ For instance, with colorectal cancer, survival falls from 91% for localized disease to 13% for distant disease, and with lung and bronchus cancers survival declines from 64.7% (localized) to 9.7% (distant).^6,7^ Despite the proven efficacy of early-stage screening programs, a substantial proportion of late-stage cancer diagnoses persist nationally, underscoring significant systemic barriers in the timely delivery of cancer care.^3,4^ The burden of advanced disease varies significantly by cancer type: lung and bronchus cancer patients comprising the highest proportion, with 45.1% of diagnoses occurring at the distant stage, compared to 23.4% for colon cancer, 18.9% for rectum cancer, and 31.0% for female breast cancer (where late-stage includes regional and distant disease).^4^ The continued diagnosis of late-stage cancers, particularly for cancers with well-established screening guidelines (e.g. colorectal, breast), reveals persistent disparities in access to and utilization of these life-saving technologies across the nation.^4,8^

Late-stage cancer diagnoses are closely associated with patient demographics and systemic socioeconomic inequities.^9,10^ Men and women living in low-income neighborhoods face a 10%-point lower 5-year cancer survival rate than their more affluent counterparts, underscoring the influence of area-level socioeconomic factors on cancer outcomes.^11^ Lower rates of screening adherence among economically vulnerable groups have also been documented.^3^ Colorectal cancer screening rates are substantially lower among uninsured persons (21%) and recent immigrants (29%) and breast cancer screening rates were the lowest among uninsured women (29%) and recent immigrants (37%).^3^ Racial and ethnic disparities in cancer survival also persist, indicating that economic factors alone do not fully account for inequities in cancer survival.^9,11^ Even after adjusting for poverty levels, African American, American Indian, and Alaskan Native men and women exhibited lower 5-year survival rates than non-Hispanic Whites.^11^

To obtain a comprehensive understanding of cancer stage disparities, the effect of the social environment also needs to be assessed, rather than relying solely on patient-level data. The Area Deprivation Index (ADI) is a validated composite measure that quantifies neighborhood-level socioeconomic disadvantage across factors associated with income, employment, education level, and housing quality and is typically aggregated to the census block group level.^12^ The ADI, often represented as a State Rank from 1 (least deprived) to 10 (most deprived) on a local scale or 1 (most affluent) to 100 (most deprived) on a national scale, ranks areas by disadvantage such that higher values reflect greater structural disadvantage across these domains.^12^ For cancer research, neighborhood disadvantage as measured by the ADI has been shown to be significantly associated with advanced-stage cancer diagnosis and subsequently worse survival, often independent of individual demographic and socioeconomic factors.^13,14^ However, much of the existing evidence is derived from population-based registries or single-site studies, with limited evaluation of how neighborhood-level disadvantage interacts with individual clinical and access-related factors within large, multi-site academic health systems.

The objective of this study was to quantify the independent associations between patient demographics, cancer type, insurance status, comorbidity burden, and neighborhood-level socioeconomic disadvantage with the likelihood of a late-stage (AJCC stage III/IV) cancer diagnosis among patients with breast, colorectal, and lung and bronchus cancer treated within a large, multi-site academic health system. Specifically, we evaluated whether neighborhood-level socioeconomic measures, including the Area Deprivation Index (ADI), Social Vulnerability Index (SVI), and Healthy Places Index (HPI), were associated with stage at diagnosis after adjustment for individual-level demographic and clinical factors. We hypothesized that patients with lung and colorectal cancers would have substantially higher odds of late-stage diagnosis compared to those with breast cancer and that residence in more socioeconomically disadvantaged neighborhoods would be associated with higher odds of late-stage diagnosis.

## Materials & Methods

### Study Design and Data Source

This study employed a retrospective cohort design utilizing patient data extracted from the University of California Data Discovery Platform (UCDDP), a HIPAA-compliant repository of standardized electronic health records from all six UC Health academic medical centers (Davis, Irvine, Los Angeles, Riverside, San Diego, and San Francisco). The UCDDP employs the Observational Medical Outcomes Partnership (OMOP) Common Data Model version 5.4, which enables standardized querying and analysis of healthcare data across multiple institutions. Data extraction occurred in October 2025. Under IRB protocol 1604619-1, the University of California Health System has granted this study an exemption from human subjects protection.

### Study Population

The final study cohort included patients over the age of 18 diagnosed with breast, colorectal, or lung and bronchus cancers that were diagnosed from January 1, 2015 to October 1, 2025. Age at diagnosis was recorded for all patients and modeled as a continuous variable in regression analyses. Cancer diagnoses were identified using International Classification of Diseases, 10th Revision, Clinical Modification (ICD-10-CM) codes mapped to standardized OMOP concept identifiers. The cancer types included breast cancer (ICD-10-CM codes C50.x), colorectal cancer (C18.x-C21.x), and lung and bronchus cancer (C34.x). Patients were excluded for having an unknown cancer type, unknown staging information, or if their residential address could not be linked to geospatial socioeconomic data.

### Primary Exposure

The primary exposure variables focused on neighborhood-level socioeconomic disadvantage, linked to each patient’s residential address at diagnosis using three validated indices. The Area Deprivation Index (ADI) State Rank is a composite measure of socioeconomic disadvantage based on 17 factors across income, employment, education, and housing quality, which ranks neighborhoods within a state such that a higher rank indicates greater deprivation.^12^ To improve interpretability and facilitate comparison across levels of disadvantage, ADI State Rank was categorized into tertiles representing low, medium, and high neighborhood deprivation based on the distribution of ADI values within the study population, with higher tertiles indicating greater deprivation. ADI tertiles were used as the primary neighborhood exposure.

In secondary analyses, the neighborhood socioeconomic context was further evaluated using two additional, validated indices. The Social Vulnerability Index (SVI) is a composite measure developed by the Centers for Disease Control and Prevention (CDC) that captures vulnerability related to socioeconomic status, household composition, minority status, and housing and transportation, with higher scores associated with greater social vulnerability.^15^ The Healthy Places Index (HPI) measures neighborhood conditions that support health, including economic opportunity, education, transportation access, housing, and environmental quality, with higher scores reflecting more favorable neighborhood health conditions and greater access to community resources.^16^ SVI and HPI were modeled as continuous variables in regression analyses to assess whether alternative dimensions of neighborhood context were independently associated with stage at diagnosis.

### Primary Outcomes

The primary outcome was the stage at diagnosis, represented as a binary outcome based on the American Joint Committee on Cancer (AJCC) staging system. Stage 0, I, and II were categorized as early-stage disease (n = 10,927; 85.9%) and served as the reference group, while stages III and IV were categorized as late-stage disease (n = 1,794; 14.1%). This outcome was selected given its established association with cancer survival and sensitivity to delays in screening, diagnostic evaluation, and care initiation.

### Secondary Outcomes

Secondary analyses compared neighborhood-level socioeconomic characteristics between patients diagnosed at early versus late-stages. Mean values of ADI, SVI, and HPI were compared across stage groups using bivariate statistical tests to characterize unadjusted differences in neighborhood context by stage at diagnosis.

### Covariates

Clinical and demographic covariates were selected based on existing literature and clinical relevance. Covariates included cancer type (categorical: breast, colorectal, and lung and bronchus, with breast cancer as the reference group), age at diagnosis (modeled as a continuous variable in years), sex (categorical: female or male, with female as the reference group), race (categorical: White, Asian, Black or African American, Native Hawaiian or Other Pacific Islander, American Indian or Alaska Native, Multiracial, Other race, and Unknown, with White as the reference group), and ethnicity (categorical: Not Hispanic or Latino, Hispanic or Latino, and Unknown).

Insurance status and comorbidity burden were also included as key covariates given their relevance to healthcare access and utilization. Comorbidity burden was measured using the Charlson Comorbidity Index (CCI), a validated weighted index that summarizes the presence of chronic conditions associated with mortality risk based on diagnosis codes, and was modeled as a continuous variable.^17^

### Statistical Analysis

Descriptive statistics for the cohort were calculated with continuous variables (i.e. age, ADI, SVI, and HPI) summarized using means ± standard deviations (SD), and categorical variables (i.e. gender, race, cancer type) reported as frequencies and percentages (n (%)).

Bivariate analyses compared patient characteristics between the early-stage and late-stage diagnosis groups using chi-square tests for categorical variables and independent samples t-tests for continuous variables. As this was a retrospective observational study, participants were not randomized to exposure groups. Blinding was not applicable, as all analyses were conducted on pre-existing, de-identified electronic health record data.

To determine adjusted associations with late-stage diagnosis, a series of sequential multivariable logistic regression models were constructed with results presented as odds ratios (OR) and 95% confidence intervals (CI). A formal a priori power calculation was not performed, as the study included all eligible patients meeting inclusion criteria during the study period. The base model (Model 1) included patient-level demographic and clinical covariates, including age at diagnosis, sex, race, ethnicity, cancer type, insurance status, and the CCI. Breast cancer, female sex, White race, and private insurance served as reference groups. Subsequent models evaluated the independent contribution of neighborhood-level socioeconomic factors. Model 2 added ADI tertiles to the base model. Model 3A added SVI as a continuous variable to the base model and Model 3B also added HPI as a continuous variable to the base model. This approach allowed for the direct comparison of neighborhood indices while maintaining a consistent set of individual-level covariates across models. Model fit and performance were assessed using Akaike Information Criterion (AIC) and pseudo-R^2^ statistics. All analyses were performed using Python (version 3.14) with the statsmodels package (version 0.14.6) and PySpark for data management.

## Results

### Sample Characteristics

The final analytic cohort consisted of 27,064 adult patients diagnosed with breast, colorectal, or lung and bronchus cancer between January 1, 2015 and October 1, 2025 across six University of California academic medical centers (Table 1). The mean age at diagnosis was 61.1 ± 13.1 years, and the cohort was predominantly female (80.2%), reflecting the large proportion of breast cancer cases. More than half of patients identified as White (55.8%), followed by Asian patients (14.4%), Black or African American patients (4.0%), and small proportions of other racial groups. Breast cancer accounted for 62.9% of diagnoses, while colorectal and lung and bronchus cancers represented 19.6% and 17.5% of cases, respectively.

Overall, 17.6% of patients were diagnosed at a late stage (AJCC stage III/IV). Insurance coverage varied substantially across the cohort. Over half of patients were covered by private insurance, nearly one-fifth were insured through Medicaid, and a notable proportion had other or unknown insurance coverage (Table 1). On average, patients resided in neighborhoods characterized by moderate socioeconomic disadvantage, as indicated by mean ADI, SVI, and HPI scores.

### Demographic, Clinical, and Insurance Factors Associated with Late-Stage Diagnosis

In multivariable analyses adjusting for demographic, clinical, insurance, and comorbidity factors, cancer type emerged as the most influential predictor of late-stage diagnosis (Table 2). Compared with breast cancer, lung and bronchus cancer was associated with nearly fourteen-fold higher odds of late-stage diagnosis (OR = 13.89; 95% CI: 12.55–15.38), while colorectal cancer was associated with approximately eight-fold higher odds (OR = 8.39; 95% CI: 7.62–9.25).

Beyond cancer type, several individual-level characteristics were independently associated with stage at diagnosis. Male sex was associated with modestly higher odds of late-stage diagnosis compared with female sex (OR = 1.09; 95% CI: 1.00–1.18). Insurance status demonstrated a significant association with diagnostic stage. Patients insured through Medicaid (OR = 1.16; 95% CI: 1.06–1.28) and those with other or unknown insurance (OR = 1.39; 95% CI: 1.26–1.52) had significantly higher odds of late-stage diagnosis compared with privately insured patients. In contrast, patients receiving care through the Veterans Affairs system experienced lower odds of late-stage diagnosis (OR = 0.76; 95% CI: 0.58–1.01).

Increasing age at diagnosis (OR = 0.99 per year; 95% CI: 0.98–0.99) and higher Charlson Comorbidity Index scores (OR = 0.83; 95% CI: 0.79–0.87) were associated with lower odds of late-stage diagnosis. This inverse association likely reflects more frequent healthcare contact, monitoring, and opportunities for cancer detection among older individuals and those with greater comorbidity burden rather than a protective effect of comorbidity itself (Table 2).

### Neighborhood Context and Late-Stage Diagnosis

After incorporating neighborhood-level socioeconomic context, residence in more deprived neighborhoods remained independently associated with late-stage diagnosis (Table 3). Relative to patients residing in neighborhoods in the lowest ADI tertile, those in the medium deprivation tertile had approximately 21% higher odds of late-stage diagnosis (95% CI), while those in the highest tertile had approximately 10% higher odds (95% CI). The stronger association observed in the medium tertile suggests a non-linear relationship between neighborhood deprivation and diagnostic delays.

Evaluation of alternative neighborhood indices yielded mixed findings. The Social Vulnerability Index was not independently associated with late-stage diagnosis after adjustment for individual-level factors (Table 4). In contrast, higher Healthy Places Index scores were significantly associated with lower odds of late-stage diagnosis (Table 5). These findings suggest that neighborhood characteristics reflecting access to health-promoting resources may support earlier detection.

### Model Performance and Comparison

Model performance metrics were similar across sequential multivariable logistic regression models, with comparable pseudo-R^2^ values indicating similar overall explanatory power (Table 6). Although models incorporating neighborhood-level indices demonstrated slightly lower Akaike Information Criterion values, these differences should be interpreted cautiously due to reduced sample sizes resulting from missing neighborhood-level data.

## Discussion

In this large, multi-site academic health system, we found that stage at cancer diagnosis was shaped by a combination of clinical, individual-level, and neighborhood-level factors, with cancer type emerging as the most influential determinant of late-stage presentation. Lung and colorectal cancers were associated with substantially higher odds of late-stage diagnosis compared with breast cancer. These findings reinforce the central role of cancer-specific biology, symptom presentation, and screening access in determining stage at diagnosis.

The significantly higher odds of late-stage diagnosis observed for lung and colorectal cancers are consistent with prior evidence demonstrating substantial variation in stage distribution, access to, and utilization of recommended screening across cancer types.^18,19^ Lung cancer, in particular, is frequently diagnosed at advanced stages due to non-specific early symptoms and historically limited screening uptake, while colorectal cancer continues to experience gaps in screening participation despite well-established guidelines.^18,19^ These persistent disparities highlight the need for enhanced early detection strategies, particularly for cancers with high late-stage burden.

Beyond cancer type, individual-level access-related factors played an important role in diagnostic timeliness. Insurance status was a strong and consistent predictor of late-stage diagnosis, with Medicaid-insured patients and those with other or unknown insurance experiencing higher odds of advanced disease at presentation compared with privately insured patients. These findings align with prior literature demonstrating association between insurance coverage, access to preventative services, and stage at diagnosis.^8,10^ In contrast, patients receiving care through the Veterans Affairs system experienced lower odds of late-stage diagnosis, suggesting that integrated healthcare delivery models with coordinated preventive and diagnostic services may mitigate delays in cancer detection. Age at diagnosis and comorbidity burden were inversely associated with late-stage diagnosis, likely reflecting greater healthcare engagement among older individuals and patients with higher comorbidity burden, who may have more frequent clinical encounters and opportunities for cancer screening or incidental detection.

Residence in more socioeconomically deprived neighborhoods, as measured by ADI tertiles, was independently associated with higher odds of late-stage diagnosis. This finding is consistent with prior studies demonstrating that neighborhood disadvantage is associated with advanced-stage cancer presentation and worse outcomes, often independent of individual socioeconomic characteristics.^13,20^ These findings suggest that structural barriers within disadvantaged environments such as transportation challenges, competing social demands, and limited access to preventative services continue to influence diagnostic timeliness.

Alternatively, individuals in the most deprived neighborhoods may be more likely to qualify for targeted public health programs or safety-net services that can mediate barriers to care. These findings highlight the complexity of neighborhood effects and suggest that deprivation does not operate uniformly across contexts.

In contrast to ADI, the Social Vulnerability Index was not independently associated with stage at diagnosis after adjustment for individual-level factors. While SVI captures aspects of vulnerability relevant to emergency preparedness and population-level risk, it may be less sensitive to barriers specific to cancer screening and diagnostic pathways within academic health systems.^15^ Conversely, the Healthy Places Index demonstrated a protective association, indicating that neighborhood characteristics reflecting access to health-promoting resources and infrastructure may facilitate earlier cancer detection.^16^ These findings suggest that the relevance of neighborhood indices may vary depending on the specific outcome of interest and the healthcare context in which they are applied. Across all models, the inclusion of neighborhood-level measures resulted in relatively minimal improvements in model performance compared with models including demographic, clinical, and insurance variables alone. This suggests that within a large academic health system, individual-level access and clinical factors explain most of the variation in stage at diagnosis, while neighborhood context provides additional context.

The strengths of the study include its large, diverse cohort drawn from multiple academic medical centers and the use of standardized electronic health record data to ensure consistently measured exposures and outcomes. Additionally, the evaluation of multiple neighborhood-level indices allowed for comparison of different dimensions of socioeconomic context. Limitations include restriction to a single academic health system, potential misclassification of neighborhood exposure based on residential address at diagnosis, missing neighborhood-level data for a subset of patients and the impact of unmeasured factors such as individual screening history, health literacy, or primary care access.

## Conclusion

Findings from this large, multi-site study confirm that the risk of a late-stage cancer diagnosis is not distributed randomly but is shaped by a combination of clinical, individual-level, and structural factors. The persistence of neighborhood-level associations after adjustment for individual demographic, insurance, and comorbidity factors suggest that the physical and social environment acts as an important structural determinant of diagnostic timeliness, underscoring the need for interventions that target community-level infrastructure and access to care.

Together, these findings suggest that efforts to reduce late-stage cancer diagnosis should prioritize cancer-specific early detection strategies, particularly for lung and colorectal cancers, while also addressing structural barriers related to insurance coverage and neighborhood disadvantage. Leveraging geospatial tools such as the ADI may help health systems and public health agencies identify communities at elevated risk for delayed diagnosis and guide targeted outreach, screening, and patient navigation efforts to advance equity in cancer outcomes.

## Figures and Tables

**Table 1.** Baseline Characteristics of the Study Population Baseline demographic, clinical, insurance, and neighborhood-level socioeconomic characteristics of 27,064 adult patients diagnosed with breast, colorectal, or lung and bronchus cancer across six University of California academic medical centers between 2015 and 2025. Continuous variables are presented as mean ± standard deviation, and categorical variables are presented as number (percentage). Neighborhood socioeconomic context was assessed using the Area Deprivation Index (ADI), Social Vulnerability Index (SVI), and Healthy Places Index (HPI). Cancer stage at diagnosis was categorized using AJCC criteria, with stages 0–II classified as early-stage and stages III–IV classified as late-stage disease.

Characteristic	Overall (N=27,064)
Total N	27,064
**Age at diagnosis, years**	
Mean ± SD	61.1± 13.1
**Gender, n (%)**	
Female	21,713 (80.2%)
Male	5,329 (19.7%)
Unknown	19 (0.1%)
Other	3 (0.0%)
**Race, n (%)**	
White	15,103 (55.8%)
Asian	3,895 (14.4%)
Other	4,026 (14.9%)
Black	1,073 (4.0%)
Pacific Islander	156 (0.6%)
American Indian or Alaska Native	88 (0.6%)
Unknown	2,723 (10.1%)
**Charlson Comorbidity Index**	
Mean ± SD	0.2 ± 0.7
**Area Deprivation Index**	
Mean ± SD	4.1 ± 2.7
**Social Vulnerability Index**	
Mean ± SD	3.0 ± 1.1
**Healthy Places Index**	
Mean ± SD	3.1 ± 1.0
**Cancer Type, n (%)**	
Breast Cancer	17,030 (62.9%)
Lung and Bronchus Cancer	4,734 (17.5%)
Colon and Rectum Cancer	5,300 (19.6%)
**Cancer Staging, n (%)**	
Early Stages	22,300 (82.4%)
Late Stages	4,764 (17.6%)
**Insurance, n (%)**	
Private Insurance	13,790 (51.0%)
Medicaid	5,074 (18.7%)
Other/Unknown	4,680 (17.3%)
Medicare Advantage	1,750 (6.5%)
Medicare	1,378 (5.1%)
Veteran Affairs	391 (1.4%)

**Table 2.** Multivariable Logistic Regression Results (Model 1): Demographic, Clinical, Insurance, and Comorbidity Factors Associated With Late-Stage Cancer Diagnosis Adjusted odds ratios (ORs) and 95% confidence intervals (CIs) from multivariable logistic regression examining associations between patient demographics (race and sex), cancer type, insurance status, age at diagnosis, and Charlson Comorbidity Index with odds of late-stage cancer diagnosis (AJCC stage III/IV). White race, female sex, breast cancer, and private insurance served as reference categories. All covariates were included simultaneously in the model. An OR greater than 1 indicates higher odds of late-stage diagnosis, while an OR less than 1 indicates lower odds.

Variable	OR	95% CI	P-Value
American Indian or Alaska Native	0.855	(0.453, 1.612)	0.628
Asian	1.109	(1.004, 1.226)	0.042
Black	1.128	(0.949, 1.341)	0.172
Pacific Islander	1.069	(0.678, 1.684)	0.774
Other Race	1.089	(0.982, 1.207)	0.105
Unknown Race	1.155	(1.024, 1.303)	0.019
White	–	–	–
Male	1.090	(1.004, 1.183)	0.039
Other Sex	0.000	(0.000, Inf)	1.000
Unknown Sex	3.238	(1.049, 9.998)	0.041
Female	–	–	–
Colorectal Cancer	8.394	(7.619, 9.248)	<0.001
Lung Cancer	13.893	(12.548, 15.383)	<0.001
Breast Cancer	–	–	–
Veteran Affairs	0.763	(0.579, 1.005)	0.055
Medicaid	1.160	(1.055, 1.275)	0.002
Medicare Advantage	1.085	(0.939, 1.255)	0.269
Medicare	1.113	(0.948, 1.306)	0.190
Other/Unknown Insurance	1.386	(1.261, 1.523)	<0.001
Private Insurance	–	–	–
Age at Diagnosis	0.986	(0.983, 0.989)	<0.001
Charlson Comorbidity Index	0.833	(0.794, 0.873)	<0.001

**Table 3.** Multivariable Logistic Regression Results (Model 2): Model 1 Plus Area Deprivation Index (ADI) Tertiles Adjusted odds ratios (ORs) and 95% confidence intervals (CIs) from multivariable logistic regression evaluating the association between neighborhood-level socioeconomic deprivation and late-stage cancer diagnosis. Area Deprivation Index (ADI) State Rank was categorized into tertiles representing low, medium, and high neighborhood deprivation, with the lowest tertile serving as the reference group. Models were adjusted for demographic characteristics, cancer type, insurance status, age at diagnosis, and Charlson Comorbidity Index. Reduced sample size reflects exclusion of patients with missing neighborhood-level socioeconomic data.

Variable	OR	95% CI	P-Value
American Indian or Alaska Native	0.803	(0.417, 1.548)	0.513
Asian	1.169	(1.055, 1.295)	0.003
Black	1.149	(0.963, 1.371)	0.124
Pacific Islander	1.015	(0.631, 1.632)	0.953
Other Race	1.075	(0.965, 1.196)	0.188
Unknown Race	1.177	(1.039, 1.332)	0.010
White	–	–	–
Male	1.107	(1.017, 1.205)	0.018
Other Sex	0.000	(0.000, Inf)	0.996
Unknown Sex	3.187	(0.876, 11.599)	0.079
Female	–	–	–
Colorectal Cancer	7.854	(7.103, 8.684)	<0.001
Lung Cancer	13.651	(12.297, 15.156)	<0.001
Breast Cancer	–	–	–
Veteran Affairs	0.718	(0.537, 0.958)	0.025
Medicaid	1.165	(1.056, 1.285)	0.002
Medicare Advantage	1.084	(0.935, 1.258)	0.284
Medicare	1.095	(0.929, 1.291)	0.279
Other/Unknown Insurance	1.313	(1.189, 1.451)	<0.001
Private Insurance	–	–	–
ADI – High Tertile	1.101	(1.008, 1.202)	0.032
ADI – Medium Tertile	1.214	(1.107, 1.331)	<0.001
ADI – Low Tertile	–	–	–
Age at Diagnosis	0.988	(0.985, 0.991)	<0.001
Charlson Comorbidity Index	0.832	(0.793, 0.874)	<0.001

**Table 4.** Multivariable Logistic Regression Results (Model 3A): Model 1 Plus Social Vulnerability Index (SVI) Adjusted odds ratios (ORs) and 95% confidence intervals (CIs) from multivariable logistic regression assessing the association between the Social Vulnerability Index (SVI) and late-stage cancer diagnosis. SVI was modeled as a continuous variable, with higher values indicating greater neighborhood-level social vulnerability. Models were adjusted for demographic characteristics, cancer type, insurance status, age at diagnosis, and Charlson Comorbidity Index. An OR of 1.00 indicates no association between SVI and stage at diagnosis after adjustment.

Variable	OR	95% CI	P-Value
American Indian or Alaska Native	0.914	(0.483, 1.729)	0.782
Asian	1.150	(1.039, 1.273)	0.007
Black	1.151	(0.964, 1.373)	0.119
Pacific Islander	1.014	(0.630, 1.630)	0.955
Other Race	1.120	(1.007, 1.246)	0.038
Unknown Race	1.182	(1.039, 1.338)	0.008
White	–	–	–
Male	1.105	(1.016, 1.203)	0.020
Other Sex	0.000	(0.000, Inf)	1.000
Unknown Sex	3.219	(0.880, 11.776)	0.077
Female	–	–	–
Colorectal Cancer	7.911	(7.157, 8.743)	<0.001
Lung Cancer	13.655	(12.304, 15.154)	<0.001
Breast Cancer	–	–	–
Veteran Affairs	0.738	(0.554, 0.984)	0.039
Medicaid	1.190	(1.078, 1.314)	<0.001
Medicare Advantage	1.103	(0.951, 1.279)	0.193
Medicare	1.125	(0.955, 1.324)	0.159
Other/Unknown Insurance	1.345	(1.218, 1.485)	<0.001
Private Insurance	–	–	–
Social Vulnerability Index	1.001	(0.966, 1.037)	0.964
Age at Diagnosis	0.988	(0.985, 0.990)	<0.001
Charlson Comorbidity Index	0.834	(0.794, 0.875)	<0.001

**Table 5.** Multivariable Logistic Regression Results (Model 3B): Model 1 Plus Healthy Places Index (HPI) Adjusted odds ratios (ORs) and 95% confidence intervals (CIs) from multivariable logistic regression evaluating the association between the Healthy Places Index (HPI) and late-stage cancer diagnosis. HPI was modeled as a continuous variable, with higher scores reflecting more favorable neighborhood health conditions and greater access to community resources. Models were adjusted for demographic characteristics, cancer type, insurance status, age at diagnosis, and Charlson Comorbidity Index. An OR less than 1 indicates lower odds of late-stage diagnosis associated with more favorable neighborhood conditions.

Variable	OR	95% CI	P-Value
American Indian or Alaska Native	0.828	(0.429, 1.600)	0.575
Asian	1.163	(1.050, 1.288)	0.004
Black	1.117	(0.936, 1.334)	0.220
Pacific Islander	1.018	(0.633, 1.637)	0.942
Other Race	1.092	(0.981, 1.216)	0.108
Unknown Race	1.175	(1.038, 1.330)	0.011
White	–	–	–
Male	1.101	(1.012, 1.198)	0.026
Other Sex	0.000	(0.000, Inf)	0.999
Unknown Sex	3.161	(0.856, 11.678)	0.084
Female	–	–	–
Colorectal Cancer	7.877	(7.125, 8.709)	<0.001
Lung Cancer	13.613	(12.263, 15.111)	<0.001
Breast Cancer	–	–	–
Veteran Affairs	0.733	(0.549, 0.979)	0.035
Medicaid	1.155	(1.047, 1.276)	0.004
Medicare Advantage	1.091	(0.941, 1.266)	0.250
Medicare	1.117	(0.948, 1.317)	0.185
Other/Unknown Insurance	1.331	(1.205, 1.470)	<0.001
Private Insurance	–	–	–
Healthy Places Index	0.957	(0.922, 0.993)	0.019
Age at Diagnosis	0.988	(0.985, 0.991)	<0.001
Charlson Comorbidity Index	0.832	(0.793, 0.874)	<0.001

**Table 6.** Model Fit and Performance Comparison Across Sequential Multivariable Logistic Regression Models Comparison of model performance metrics across sequential multivariable logistic regression models, including sample size (N), Akaike Information Criterion (AIC), and pseudo-R^2^. Model 1 included demographic, clinical, insurance, and comorbidity variables; Model 2 additionally included Area Deprivation Index (ADI) tertiles; Model 3A included Social Vulnerability Index (SVI); and Model 3B included Healthy Places Index (HPI). Differences in sample size across models reflect missing neighborhood-level socioeconomic data. Lower AIC values indicate improved model fit, though comparisons should be interpreted cautiously due to differing sample sizes.

Model	N	AIC	Pseudo R^2^
1: Demographics + Insurance + Comorbidity	27,063	20559.73	0.1872
2: Model 1 + ADI	25,780	19372.54	0.1857
3A: Model 1 + SVI	25,940	19533.48	0.1846
3B: Model 1 + HPI	25,784	19392.23	0.1847

## Data Availability

This study utilized data from the University of California Data Discovery Platform (UCDDP), a HIPAA-limited dataset comprising patient records from the six UC Health academic medical centers. To protect patient confidentiality, the data are not publicly accessible. The data used in this study are not publicly available due to HIPAA and institutional data use agreements but may be accessed through the University of California Data Discovery Platform following appropriate approvals.
